# Chemical profile of *Tetraclinis articulata* (Vahl) Masters, and *Juglans regia* L. and *Olea europaea* L. var. *Sylvestris* used against oral diseases: in vitro analysis between polyphenolic content and aqueous extraction optimization

**DOI:** 10.1016/j.heliyon.2021.e07118

**Published:** 2021-05-31

**Authors:** Hazim Harouak, Jamal Ibijbijen, Laila Nassiri

**Affiliations:** Environment & Valorization of Plant and Microbial Resources Unit, Moulay Ismail University of Meknes, Faculty of Sciences, B.P 11201, Meknes, Morocco

**Keywords:** *Juglans regia* L., *Europaea* L. subsp. *europaea* var. *sylvestris*, *Tetraclinis articulata* L., Total polyphenolic compounds, Optimization of aqueous extraction, PCA analysis

## Abstract

**Objective:**

Optimization of aqueous extraction to extract the maximum amount of polyphenolic compounds that are used to treat oral disorders.

**Methods:**

Using revelation tests for phytochemical screening, Folin-Ciocalteu reagent for total phenols, catechin standard for total flavonoids, acidified vanillin for total condensed tannins, and PCA analysis to detect different correlations between plants and between employed extractions.

**Results:**

The highest (p < 0.0001); total flavonoides (195,80 ± 2,91 mg CE/g d.e) was obtained from decocted extract of *Olea europaea* L. subsp. *europaea* var. *sylvestris*, total phenolic (167,71 ± 12,52 mg GAE/g d.e) and total condensed tannins (250,44 ± 10,18 mg CE/g d.e) was obtained respectively from soxhlet extract and infused extract of *Tetraclinis articulata* L. whereas; The correlation analysis using Principal Component Analysis (PCA) was positive between infusion and decoction, between total flavonoids and total phenols which is not correlated with total condensed tannins.

**Conclusion:**

*Juglans regia* L. bark contain a higher level of polyphenolic constituents than leaves, Decoction extraction of *Olea europaea* L. var. *sylvestris* leaf recommended to increase the yield of polychenolic extracts, condensed tannins of *Tetraclinis articulata* L. are degradable in high temperature.

## Highlights

• Walnut barks are richer in polyphenolic compounds than leaves.
• Barbary Thuya leaf is richer in degradable condensed tannins at high temperatures.
• Wild olive leaf is richer in flavonoids (195.80 mg EC/g of dry decocted extract).
• Condensed tannins are uncorrelated with phenols and flavonoids.
• Flavonoids are the major polyphenolic constituents.

## 1 Introduction

Among the best preventive means against infectious and degenerative disorders, the biological activity of polyphenolic compounds (Petti and Scully, 2009).

Flavanone such as pinocembrin or pinostrobin and chalcones like 2,4 dihydroxychalcone or 2,4-dihydroxy-3-methoxychalcone, are among the polyphenolic compounds which showing a good antimicrobial activity (Ávila et al., 2008; Batovska et al., 2009), flavonols named izalpinin, rhamoncitrin and galangin, have revealed a fungicidal activity against *Trichophyton mentagrophytes, Trichophyton rubrum* and *Microsporum gypsum* (Agüero et al., 2010).

In dentisetry, Polyphenols have the ability to inhibit the formation of mixed biofilms on orthodontic surfaces (Farkash et al., 2019), Flavonols of the flavonoid family have been shown to have a positive effect against gram-positive bacteria such as (*Staphylococcus aureus, Actinomyces naeslundii* and *Lactobacillus acidophilus*), gram-negative bacteria as (*Prevotella oralis, Porphyromonas gingivalis and Prevotella melaninogenica*)*,* and *Fusobacterium nucleatum* (Cushnie et al., 2007), Luteolin, also known as a flavonic component of perilla seed (*Perilla frutescens Britton* var*. Japonica Hara*) showed the strongest antimicrobial effect among other phenolic compounds against cariogenic *streptococci* and *Porodyromonas gingivalis* (Yamamoto and Ogawa, 2002).

It has also been reported that tannins obtained from the bark of *Phyllanthus columnaris* inhibited the growth of all oral pathogens tested (Othman et al., 2019).

*Tetraclinis articulata* L. (Cupressaceae), locally called "Al'Araar" or "Azouka" in tachlhit, is among the most popular herbs, widely used in folk medicine (Montanari, 2014), is a herbaceous plant originally from the southwest of the Mediterranean, mainly from Maghreb vegetation, given its richness in essential oils that give it many biological activities it is therefore considered among the most widely used aromatic and medicinal plants in the world (Polunin and Huxley, 1967; Nicolás et al., 2004).

*Olea europaea* L., belonging to the Oleaceae family, it is among the oldest cultivated trees in the world and is a typical crop of the Mediterranean area (Scognamiglio et al., 2012).

The subsp. *europaea* var. *sylvestris* of Oleaster, is widely found along the coastal and semi-coastal areas of Mediterranean and is evergreen, this vegetation is mainly composed of sclerophyllous shrub formations that can tolerate the arid conditions that characterize the Mediterranean region (Baratta and Barbera, 1981; de Graaff and Eppink, 1999).

*Juglans regia* L. (family Juglandaceae) The Persian or common walnut is the world's most widely distributed deciduous tree species found mainly in temperate zones and is being cultivated commercially in the western and southern of America, in central and southern of Europe and in Asia (Anonimous, 1999).

The traditional use of these three trees (*Juglans regia* L., *Olea europaea* L. subsp. *europaea* var. *sylvestris* and *Tetraclinis articulata* L.) against oral affections has been reported (Harouak et al., 2018).

Our study aims to evaluate the polyphenol content of the trees studied and to compare it in terms of the aqueous extraction used.

## 2 Materials and methods

### 2.1 Plant materials

**Figure 1:**
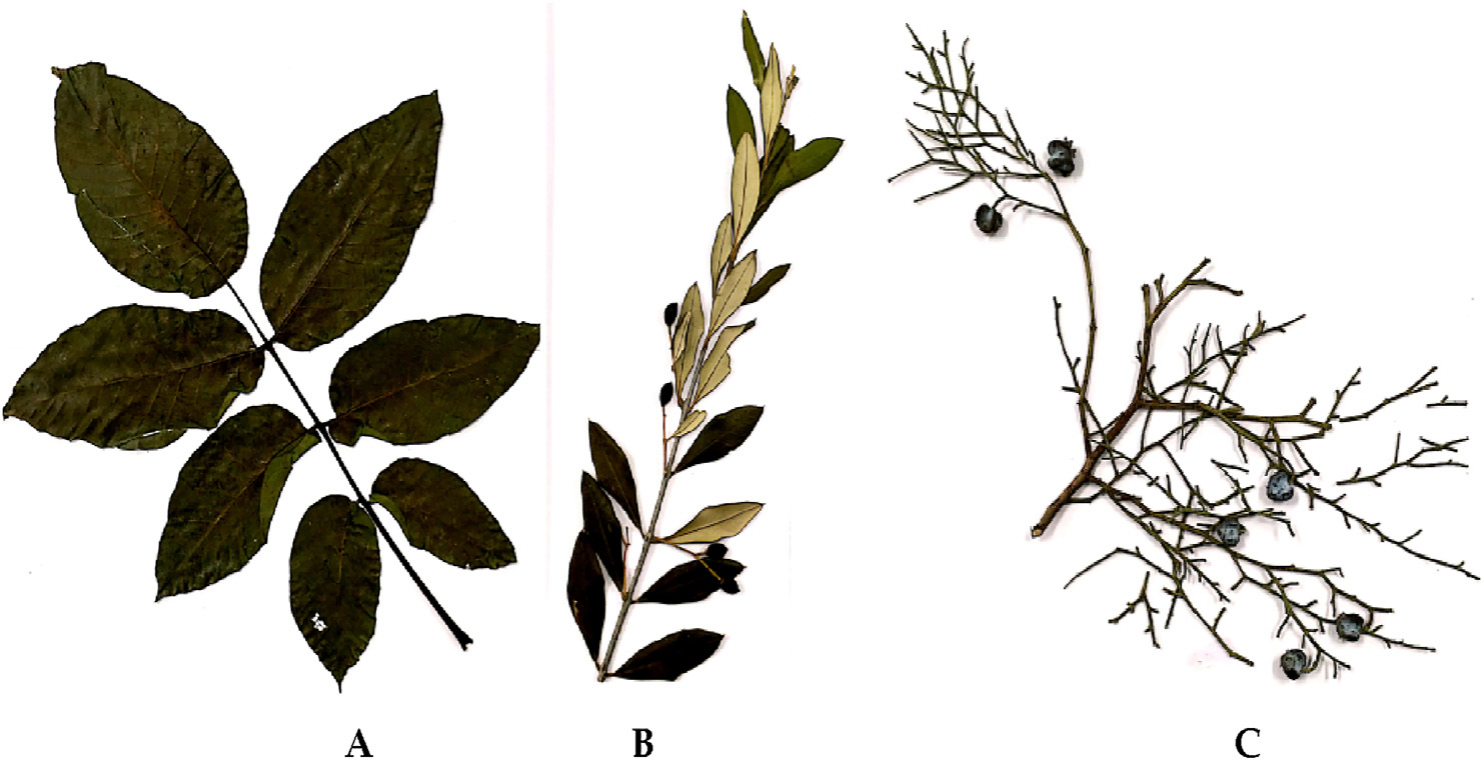
Species pictures (A: *J. regia* L.; B: *O. europaea* L. var. *Sylvestris*; C: *T. articulata* (Vahl) Masters).

Species leaves (Figure 1) and walnut barks were collected during the flowering season from with a code that has been assigned to each species:

34°03′52.9″N 5°30′09.5″W in 23/06/2019 for *Olea europaea* L. var*. Sylvestris* “OE” (MICSOLENV-OE/N°06);

33°52′10.8″N 5°32′35.3″W in 07/07/2019 for *Juglans regia* L. “JR” (MICSOLENV-JR/N°07);

33°52′39.4″N 5°54′54.9″W in 03/05/2019 for *Tetraclinis articulata* (Vahl) Masters “TA” (MICSOLENV-TA/N°15);

In our laboratory greenhouse; the plants were air-dried at room temperature and then passed through a 0.5 mm sieve in a mill; our samples were then stored in plastic bags in a dark and dry condition.

### 2.2 Moisture content

The high moisture content is closely related to the degradation of phytochemicals, moreover it is a challenge for storage and transport; therefore, and as an important first step in the process of retention of phytochemicals and subsequent processing, the removal of moisture by drying is mandatory (Wojdyło et al., 2014; Tran et al., 2020).

The water content of our plant material has been expressed as a percentage (%) according to the following formula Eq. (1) (Mohammadpour et al., 2019):

(1) MC (%) = (M1 - M2)∗100/M1

with: MC: moisture content given in percentage (%); M1: sample weight in grams after harvesting (fresh matter); M2: sample weight in grams after drying (dry matter) (Mohammadpour et al., 2019).

### 2.3 Extraction method

Aqueous extracts were prepared, 10% of each sample in distilled water Using infusion, decoction and Soxhlet techniques, after the obtained mixtures were filtered with Wathman filter paper and the recovered filtrate was evaporated in the oven at 45 degrees Celsius.

**Infusion**: to the boiling distilled water the vegetable material is added, then the mixture is left under stirring for 15 min.

**Decoction**: the plant material with distilled water and heated under stirring until it boils, then the mixture is left to stand under stirring for 15 min.

**Soxhlet technique**: the extraction is done 4 h (12 cycles) in a Soxhlet apparatus for each sample.

The percentage of extraction yields was determined by the following formula Eq. (2) (Alara et al., 2018).

(2) % Extracts Yields = (We∗100)/Wt

where W_e_ is the extract weight of plant sample (w) and W_t_ is the weight of dried plant sample.

### 2.4 Statistical analysis

A statistical analysis of collected data was carried out to highlight the significant differences between the yields of the three kinds of extraction used and investigated species, through the use of GraphPad prism 8.2.263 software, by two-way analysis of variance (ANOVA), then Tukey test to detect the degree of significance, this significance is taken at the probability of ∗p < 0.05 for a significant difference, ∗∗p < 0.01 for moderately significant difference, ∗∗∗p < 0.001 for a highly significant difference, ∗∗∗∗p < 0.0001 for a very highly significant difference (a high value of F means that something is significant, while a small value of p means that all results are significant, the null hypothesis (H0) is rejected from p < 0.05).

### 2.5 Principal Component Analysis (PCA)

PCA is a component analysis, it creates factors in the form of principal axes which are linearly related to the initial variables, which are independently of each others and hierarchically ordered, these axes are the "expression of general processes directing the distribution of several phenomena which are correlated with each other" (Béguin and Pumain, 2000), this technique transforms variables linked to each other (correlated) into new variables decorrelated from each other. These newly created variables are termed "principal components", or main axes.

To highlight different correlations between the variables (dosed polyphenolic compounds) and extraction types between the individuals (studied plants); the projection of the variables in F1 and F2 plan, were obtained using XLSTAT 2016 (18.02.01).

### 2.6 Phytochemical screening

The extracted samples were screened phytochemically to detect the presence of tannins using iron chloride, flavonoids following cyanidine reaction, terpenoids using Liebermann Burchard reaction, alkaloids according to Dragendorff and Mayer reagent, reducing sugar based on Fehling reagent (Karumi et al., 2004).

The revelation of saponins was observed in a test tube containing the extract with distilled water, the mixture was stirred for 20 s and left to stand for 15 min, the appearance of a persistent moss (more than 1 cm in height) means the presence of saponosides (Bruneton, 2009).

### 2.7 Determination of polyhenolic content

#### 2.7.1 Total phenols

The total phenolic content (TPC) was determined according to the procedure reported by Slinkard and Singleton (1977).

In test tubes for each extract (concentration of 1 mg/ml in distilled water), 100 μl were added to 4.5 ml of D.W (distilled water) and then to 100 μl of Ciocalteu-Folin reactive. This combination was then left for 3 min at ambient temperature, and then 300 μl of Na_2_C0_3_ (2% in water) has been added. After 1h30 of incubation in a dark chamber at ambient temperature, the absorbance was recorded with a spectrophotometer (UV-2005) at 760 nm against a blank containing distilled water. Under similar operating procedures, a calibration curve was performed by gallic acid (standard phenolic compound) from 100 to 1000 μg/ml; the results were presented in mg gallic acid equivalent (AGE)/1 g dry extract and the analysis was expressed as a mean of three replicates ± the standard deviation.

#### 2.7.2 Total flavonoids

To evaluate the total flavonoid concentration (TFC), we employed the approach described by (Zilic et al., 2011). A solution composed of (500 μl) of each extract (1 mg/ml) and 75 μl of NaNO_2_ (5%) was prepared. After 6 min, 150 μl of an AlCl_3_ solution (10%) was added, then the whole was left to stand at room temperature for 5 min, then 500 μl of NaOH (1M) was added and the total volume of tubes is then completed to 2.5 ml by adding distilled water.

At 510 nm and against a blank with distilled water as extraction solvent, we measured the absorbance. With same operating conditions, a calibration curve was traced using catechin as flavonoid standard reference (50–500 μg/ml); results were expressed in mg catechin equivalent (CE)/1g dry extract and the analysis was expressed as a mean of three replicates ± the standard deviation.

#### 2.7.3 Total condensed tannins

Based on the method outlined by (Broadhurst and Jones, 1978), the condensed tannin concentration using acidified vanillin was determined as follows; 0.5 ml from each sample (1 mg/ml) was mixed with 1.5 ml of vanillin reagent (4%, w/v, vanillin in methanol). After passing through the vortex, 750 μl of concentrated hydrochloric acid was added. After a second shaking of the vortex, the mixture was left in the dark at 20 °C for 15 min. The absorbance was taken at 500 nm, using a distilled water blank as extraction solvent. Under the same operating conditions, a calibration curve was plotted using catechin as the reference condensed tannin (100–1000 μg/ml); the results were expressed in mg catechin equivalent (CE)/1 g of dry extract and the mean of the analysis was calculated over three replicates ±standard deviation.

**Figure 2:**
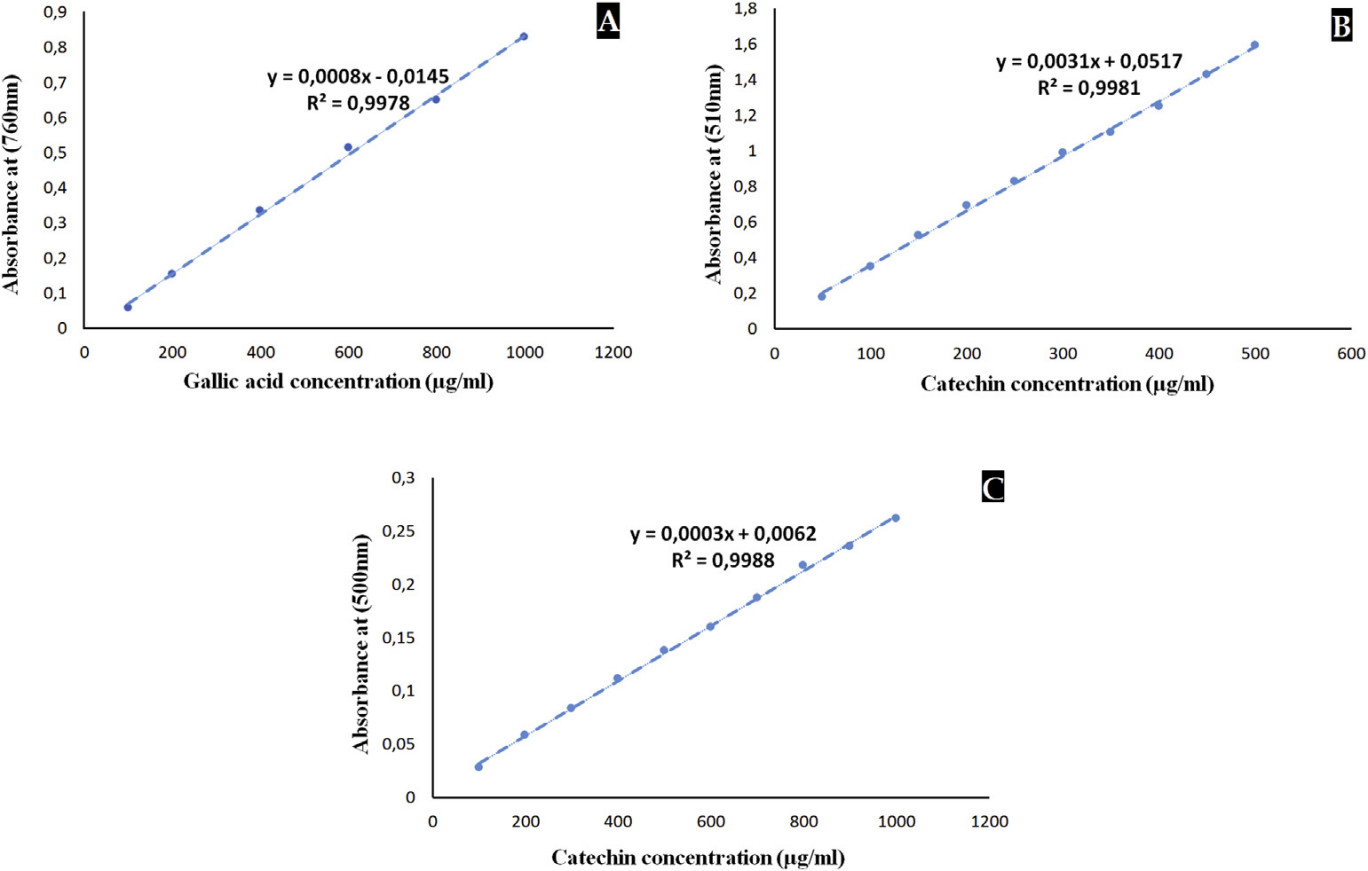
Calibration curves of gallic acid (fig A: total phenols) and catechin (fig B: total flavonoids and fig C: total condensed tannins).

TPC, TFC and TCT was obtained using the following relation Eq. (3):

(3) (c∗V)/m

with c: is the concentration of TPC, TFC or TCT from the equation of calibration curve (Figure 2), V: is the total volume of solvent (distilled water) used in the test (ml), and m represents the weight of the dried sample used (g) (Alara et al., 2018).

## 3 Results and discussion

### 3.1 Moisture content

Watter content ranged between 35.02 and 59.74% (Table 1), walnut leaves are those that contain more water which is due to its larger morphology, in fact the climatological difference at the time of harvest allows the change of temperature which is the only variable that determines the concentration of water in the air in the internal intercellular spaces; in fact when the leaf receives radiation, its temperature rises, which increases the concentration of water in these leaves (Jones, 1990), that concentration can affect phytochemical content of species; in fact it was reported that the essential oil content of *Eucalyptus citriodora* leaves dried in the shade for one week is 1.70% against 1.14% for fresh leaves (Singh et al., 1977), the increase of this content during drying period suggests the continuity and acceleration of the biosynthesis of these phytochemicals compounds after harvesting the plant (Silou et al., 2002).

**Table 1:** Moisture content percentage.

Species	*Juglans regia* L. leaves	*Juglans regia* L. barks	*Olea europaea* L. var. *Sylvestris*	*Tetraclinis articulata* (Vahl) *Masters*
Moisture Content (%)	59,74	49,74	35,02	45,71

Plants generally react to environmental aggressions by producing polyphenols and especially flavonoids, which are a means of defense of the plant species against these aggressions. Moreover, the phenolic compounds of a given species depend on several factors; intrinsic (genetic) and extrinsic (environmental) (Young et al., 1997; Carpenter and Smith, 1975).

Natural drying after harvest period is a free method of conservation, effective and which respects the environment, Indeed, this technique does not generate waste or polluting discharges. In addition, it allows plants to preserve their active principles, to improve the quality of the products by avoiding their contamination, to lower their weight in view to facilitate their transport, to multiply their use and increase their lifetime (Aghfir et al., 2007; Ankila, 2007).

### 3.2 Extractions yield

**Figure 3:**
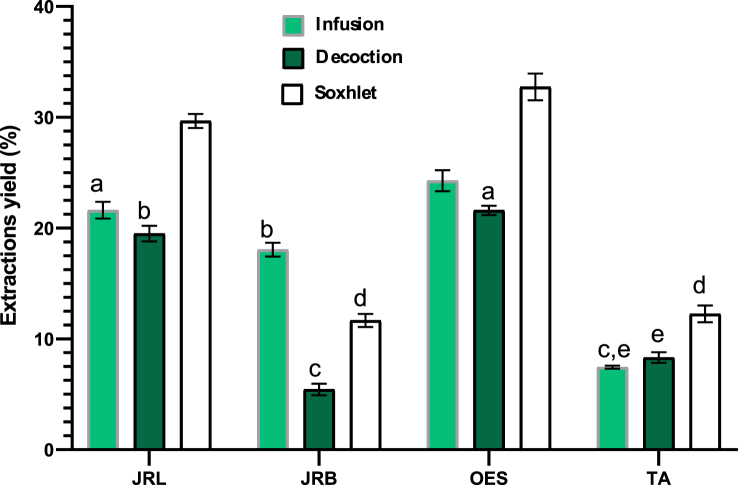
Yield extractions percentage, Values sharing same letters are not significantly different (p > 0.05), JRL = *Juglans regia* L. leaves; JRB = *Juglans regia* L. barks; OES = *Olea europaea* L. var. *Sylvestris*; TA = *Tetraclinis articulata* (Vahl) Masters.

The yields found (Figure 3) reveals that the wild olive tree is the plant that gives more yield compared to the 2 other plants, walnut leaves give more yield in aqueous extraction than bark, the comparison in terms of extraction type favors extraction by Soxhlet in a significant way (p < 0.05) except in case of walnut bark or infusion is the profitable extraction.

Walnut yields were less than some aqueous extracts of 5 cultivars (*Juglans regia* L.) green husks which are very similar in different varieties of walnut, from 31.63 to 33.69% (Oliveira et al., 2008), but it was higher than Leaf yield of ethanol:water (4:6) extract which was 20.1% (Almeida et al., 2008) and bark yield of infused extract 11.8 % (Amirou et al., 2018).

Our yields of *T. articulata* are less than those found by organic solvent extraction such as methanolic extraction 17.64 % (Dane et al., 2016) or by ethanol (60%) obtained at 240 min (Bensebia et al., 2021).

However; our pilot plant in terms of yield *Olea europaea* L. var. *Sylvestris* was very profitable compared to another study of the crude extract which gave only a maximum yield which did not exceed 6,94% (Ghania et al., 2014).

The Soxhlet in our study that extracts more which is also called continuous hot extraction is the most efficient technique for extracting vegetable oil (Teixeira et al., 2018) This type of extraction that maintains a relatively high extraction temperature with the heat of distillation flask (Tandon and Rane, 2008), it is therefore well applied, due to its high performance compared to other conventional extraction methods (Luque de Castro and García-Ayuso, 1998).

Therefore, the performance of any type of extraction depends on many parameters: extraction temperature, time, type of solvent and volume used (Chew et al., 2011; Costa et al., 2012).

According to this approach our yields can be explained by the pressure, the high temperature and the total exhaustion of vegetable matter, which is the case of Soxhlet extraction.

### 3.3 Phytochemical screening

Most of the secondary metabolites were detected by phytochemical screening; all samples contain combined tannins, flavonoids, terpenoids, reducing and antracenic compounds, while alkaloids are absent for studied simple; but *Juglans regia* reveals more flavonoids compounds than *Tetraclinis articulata* and *Olea europaea* var. *Sylvestris,* Free anthracenes and coumarins are only detected in Walnut leaf (Table 2).

**Table 2:** Phytochemical screening results.

Compounds		Species			
		*Juglans regia L.* leaves	*Juglans regia L.* barks	*Tetraclinis articulata (Vahl) Masters*	*Olea europaea L.* var. *Sylvestris*
Tannins	**Tannins** Catechic tannins Gallic tannins	+++ +++ 0	+++ +++ 0	++ + 0	+++ ++ 0
	**Alkaloids**	0	0	0	0
Flavonic Compounds	Anthocyanins Flavones Flavanones Flavanols and Flavanonols Genines Leucoanthocyans Catechols	++ 0 0 +++ +++ 0 +++	+ 0 0 0 ++ 0 +++	0 0 0 0 0 0 +++	0 0 0 0 0 0 ++
Terpenoids	Sterols and Triterpenes Saponosides	++ ++	++ +	+++ +++	+++ +++
Reducing Compounds	Reducing Compounds Mucilages Oses and holosides Cyanogenetic compounds	++ 0 0 0	+++ ++ + +	+++ +++ +++ 0	+++ 0 0 +
Anthracene derivatives	**Free anthracenes**	+++	0	0	0
	**Combined Anthracenic:** C-heterosides O-heterosides	+ ++	+ ++	+ +++	+ 0
	**Coumarins**	++	0	0	0

+++: Very positive reaction; + +: Moderately positive reaction; +: Weakly positive reaction; 0: Total absence.

A phytochemical study of: *Tetraclinis articulata* essential oil shows the presence of terpene hydrocarbons which represent 23.6% and oxygenated terpenes 28.3% (Fatiha et al., 2015), *Olea Europaea* leaf from Algeria revealed the presence of certain active substances such as flavonoids, saponins and steroids (Nora et al., 2012).

The comparison of our secondary metabolites in walnut shows that leaf are richer in flavoindes contrary to the bark which is richer in reducing compound, Other research reveals the presence in walnut leaves of tannins, flavonoids, alkaloids, phenolic compounds, carbohydrates, cardiac glycosides, proteins and steroids (Shah et al., 2013); caffeic acid, essential fatty acids, paracomaric acid and ascorbic acid. The main flavonoids present in the nut leaves are quercetin galactoside and pantocidal derivatives of quercetin, quercetin arabinoside, quercetin xyloside and quercetin rhamnoside (Mohammadi et al., 2012), bark contains reducing sugars, amino acids, flavonoids, alkaloids, tannins and phenols, steroids, saponins (NirmlaDevi et al., 2011).

Indeed; the observed difference can be explained by the fact that the synthesis of secondary metabolites in the same species is affected by many factors, like internal genetics evolution as regulated genes and/or enzymes, or by external environmental influences like light, temperature, water, salinity, etc. (Li et al., 2020).

### 3.4 Quantitative distribution of polyphenols

#### 3.4.1 Total phenolic content (as presented in Figure 4.A)

The interaction between studied samples and extraction type is considered extremely significant with:

**Figure 4:**
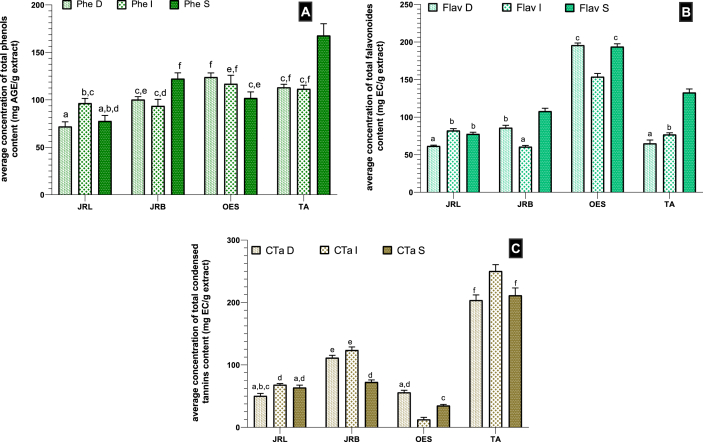
Mean concentrations of polyphenolic components in JRL = *Juglans regia* L. leaves; JRB = *Juglans regia* L. barks; OES =: *Olea europaea* L. var. *Sylvestris*; TA = *Tetraclinis articulata* (Vahl) Masters (fig A: Phe = Phenols, fig B: Flav = Flavonoids, fig C: CTa = Condensed tannins; I=Infusion, D = Decoction and S=Soxhlet extracts), Values with same characters are not significantly different (p > 0.05).

F = 30.32. DFn = 6, DFd = 24, the P value is <0.0001; also, the difference is also extremely significant between plants F = 88.22. DFn = 3, DFd = 24, the P value is <0.0001; same difference is showed between the types of extraction concerning content of total phenols F = 18.87. DFn = 2, DFd = 24, the P < 0.0001.

Studied samples contain a phenol content values not significant (p > 0.05) between plants and between extracts following Tukey's multiple comparison; except Soxhlet extract of *Tetraclinis articulata* which is very highly significant (p < 0.0001) more richer in those compounds than other two extraction type.

The finding results of our phenol concentration for walnut are highest compared to others who have found for 5 cultivars (*Juglans regia* L.) green husks; from 32.61 mg/g in cv. Mellanaise to 74.08 mg/g in cv. Franquette of gallic acid equivalent (Oliveira et al., 2008), but lower than some other research who found in ethanol/water (40%/60%) leaf extract 270 ± 3 mg GAE/g of lyophilised extract (Almeida et al., 2008), or to the macerated acetone extract (327.972 ± 0.06 μgEAG/mgE) (Boulfia et al., 2020).

While our aqueous extract contains high phenolic quantum which exceeds 150 mg/g extract compared to the fractions of *T. articulata*; ethyl acetate which contains 93.1 mg/g of extract, aqueous extract (21.2 mg/g of extract) and butanol (43.87 mg/g of extract) (Rached et al., 2018) or the one obtained with 60% ethanol (TPC (95.28 mg EAG/g)) for an extraction time of 210 min (Bensebia et al., 2021).

Also our olive leaf contain more phenol content compare to others (Talhaoui et al., 2014) who found 30 phenolic compounds represents a concentration from 52.12 to 60.64 mg/kg.

Phenol content differs from one study to another depending on the extraction type; it should be noted that many authors agree that, in order to improve both the solute solubility and diffusion coefficient, it is necessary to increase the extraction temperature and time; this procedure can degrade certain quantities of phenolic compounds (Yilmaz and Toledo, 2006; Pinelo et al., 2005).

#### 3.4.2 Total flavonoids content (as presented in Figure 4.B)

The interaction between plants and extraction type is considered extremely significant with: F = 112.37. DFn = 6, DFd = 24, the P value is <0.0001; also, the difference between plants, as well as between the extraction types is extremely significant, respectively with: F = 1975.98. DFn = 3, DFd = 24, the P value is <0.0001, and: F = 351.31. DFn = 2, DFd = 24, the P value is <0.0001.

The quantity of total flavonoids reveals values ranging from significant (p < 0.05) to extremely significant (p < 0.0001) between plants and between extracts, OES is the species which contains more flavonoids compared to other studied species which contain on average the same quantity of these interesting compounds, extraction type is very remarkable in TA which the Soxhlet extract has a greater content of flavonoids very highly significant (p < 0.0001).

The yields of Soxhlet extraction of flavonoids were higher than those of infusion or decoction in the present study, this may be explained by the fact that the increased temperature accelerates mass transfer and improves the extraction yield (Wang et al., 2008), This could be the reason why the flavonoid yield was significantly increased when the temperature was changed from of 40–60 °C and with increasing pressure to a certain value (Bimakr et al., 2011).

#### 3.4.3 Total condensed tannins content (as presented in Figure 4.C)

The interaction between plants and used extraction is considered very highly significant with: F = 44.50. DFn = 6, DFd = 24, the P value is <0.0001; also, between plants as well as between the types of extraction, the difference is extremely significant with, respectively: F = 1756.11. DFn = 3, DFd = 24, the P value is <0.0001, and: F = 27.85. DFn = 2, DFd = 24, the P value is <0.0001.

*Tetraclinis articulate* is the plant which contain more condensed tannins extracted by infusion with a very highly significant (p < 0.0001) compared to other extraction type and other studied species.

*Juglans regia* bark are rich in these compounds compared to leaf.

Thus, it should be pointed out that the tannin content can be influenced by the location where the plants were grown (Antonelli-Ushirobira et al., 2004).

### 3.5 Principal Component Analysis (PCA)

The PCA aimed to reduce the size of collected data into a smaller number of components in order to examine the grouping in quantity of phenolic compounds according to the different extraction types and studied plants.

**Figure 5:**
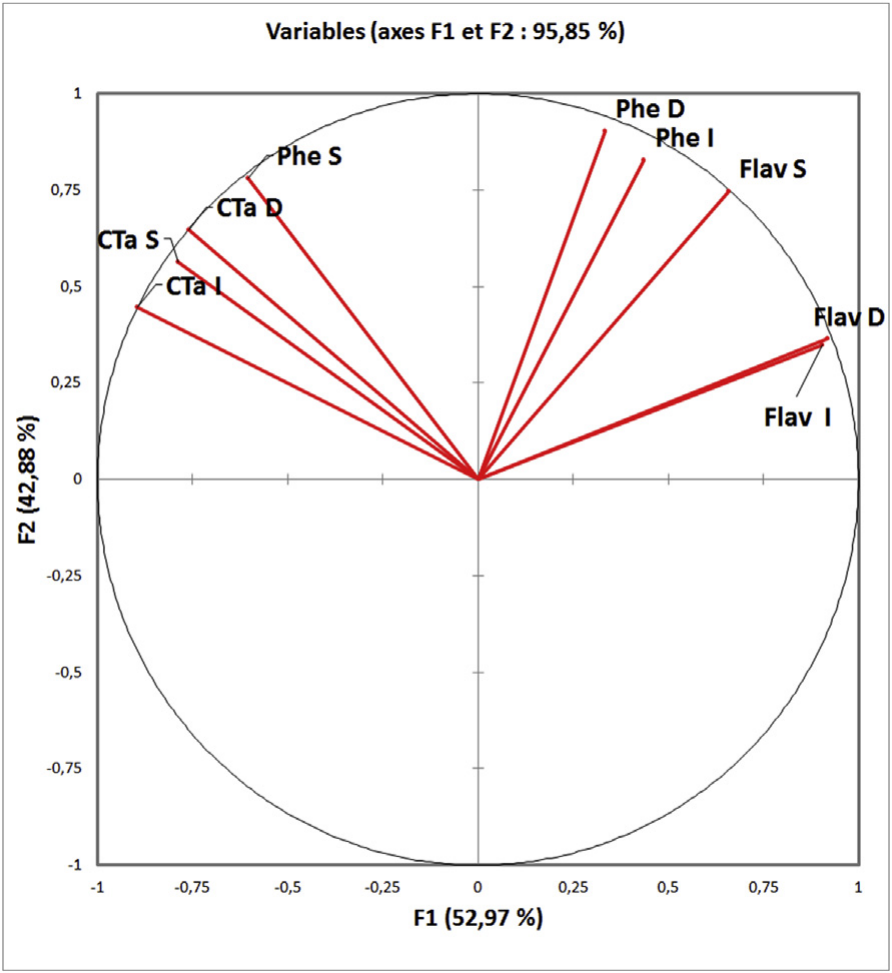
Correlation circle of variables (Phe: Phenols, Flav: Flavonoids, CTa: Condensed tannins, I: infusion, D: Decoction, S: Soxhlet).

The F1 axis represents (52.97%) and F2 (42.88%) of information; On the correlation circle (Figure 5), the variables are well presented on the F1 and F2 plane which explains 95.85% of the variability.

FlavD and FlavI are not correlated with PheS, also FlavS isn't correlated with CTaI; CTaS are strongly positively correlated with CTaD which is correlated positively with CTaI and PheS; FlavD, FlavI, FlavS, PheI and PheD are also correlated positively; The positive correlation between flavonoids and total phenols can be explained by the reason that flavonoids are the majority phenolic compounds; which is confirmed by the relationship related phenolic compounds with flavonoids content (Maisuthisakul et al., 2008).

**Figure 6:**
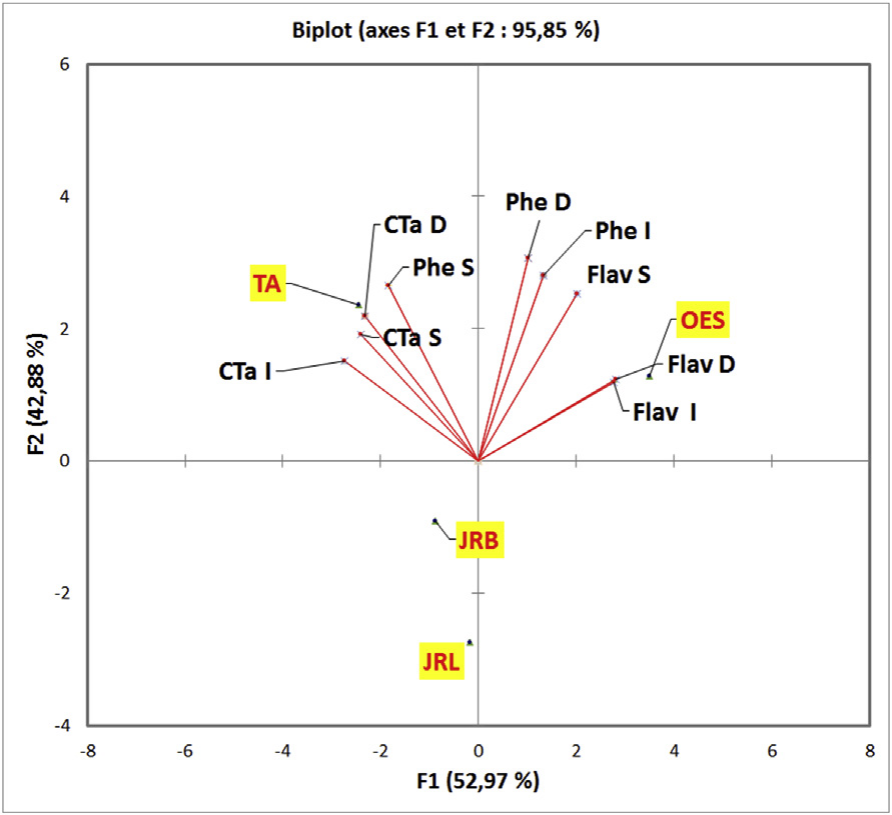
Projection of individuals on the factorial plane (F1 x F2).

Indeed, Oleaster var. *Sylvestris* tree is richer in flavonoides extracted by decoction and Soxhlet, *Tetraclinis articulata* (Vahl) Masters contain the higher quantity of condensed tannins in his infused extract and *Juglans regia* L. bark contain greater amounts of polyphenolic components than leafs (Figure 6).

## 4 Conclusion

To increase the content of polyphenolic compounds in the aqueous extracts of a given plant, it is necessary and recommended to take into consideration the plant used, its studied parts, the extraction conditions such as temperature, time and pressure.

In our study, it was clear that the bark of *J. regia* is richer in these compounds than leaves, the extraction by Soxhlet is more profitable at the level of *T. articulata*, while the decoction of *O. europaea* L. var. *sylvestris* leaves is the type of extraction that gave more polyphenolic compounds.

## Declarations

### Author contribution statement

Hazim Harouak: Conceived and designed the experiments; Performed the experiments; Analyzed and interpreted the data; Wrote the paper.

Jamal Ibijbijen: Contributed reagents, materials, analysis tools or data.

Laila Nassiri: Conceived and designed the experiments; Analyzed and interpreted the data; Wrote the paper.

### Funding statement

This research did not receive any specific grant from funding agencies in the public, commercial, or not-for-profit sectors.

### Data availability statement

Data included in article/supplementary material/referenced in article.

### Declaration of interests statement

The authors declare no conflict of interest.

### Additional information

No additional information is available for this paper.
